# Strain imaging using cardiac magnetic resonance

**DOI:** 10.1007/s10741-017-9621-8

**Published:** 2017-06-15

**Authors:** A. Scatteia, A. Baritussio, C. Bucciarelli-Ducci

**Affiliations:** 1 Cardiac Magnetic Resonance Unit, Bristol Heart Institute, NIHR Bristol Biomedical Research Centre, University of Bristol, Bristol, UK; 2 Division of Cardiology, Ospedale Medico-Chirurgico Accreditato Villa dei Fiori, Acerra, Naples, Italy

**Keywords:** Myocardial deformation imaging, Myocardial strain, Cardiovascular magnetic resonance, CMR tagging, Feature tracking

## Abstract

The objective assessments of left ventricular (LV) and right ventricular (RV) ejection fractions (EFs) are the main important tasks of routine cardiovascular magnetic resonance (CMR). Over the years, CMR has emerged as the reference standard for the evaluation of biventricular morphology and function. However, changes in EF may occur in the late stages of the majority of cardiac diseases, and being a measure of global function, it has limited sensitivity for identifying regional myocardial impairment. On the other hand, current wall motion evaluation is done on a subjective basis and subjective, qualitative analysis has a substantial error rate. In an attempt to better quantify global and regional LV function; several techniques, to assess myocardial deformation, have been developed, over the past years. The aim of this review is to provide a comprehensive compendium of all the CMR techniques to assess myocardial deformation parameters as well as the application in different clinical scenarios.

## Introduction

**Fig. 1:**
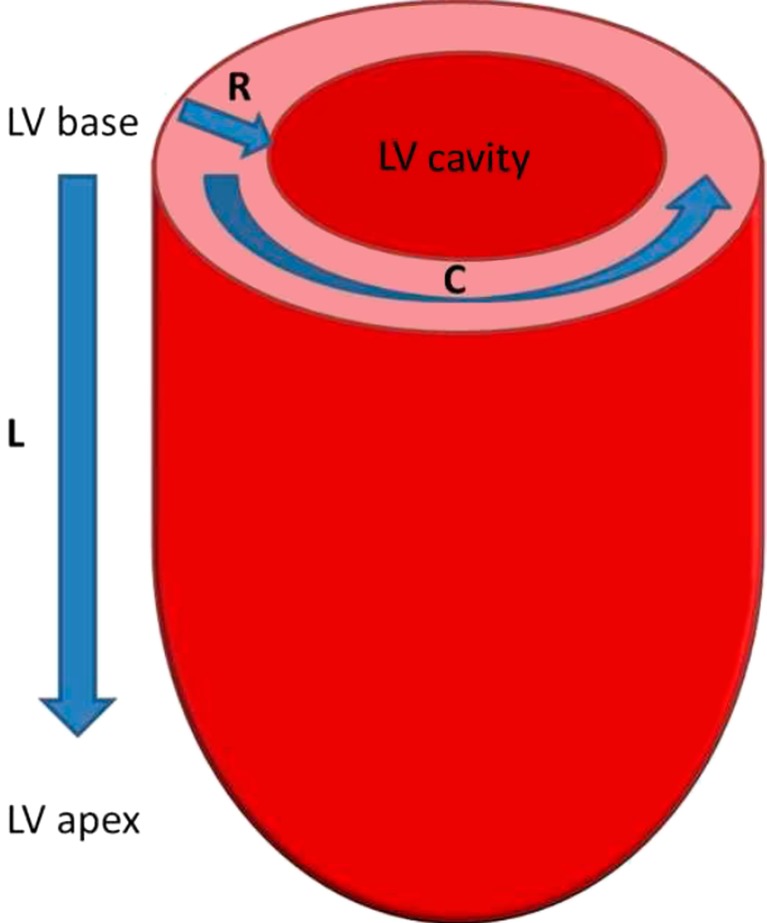
Schematic picture representing the left ventricle (LV) and the myocardial deformation directions. *L* longitudinal shortening, *C* circumferential shortening, *R* radial thickening

Myocardial deformation imaging has shown to detect early contractile dysfunction in a number of cardiovascular diseases. In an effort to better characterize biventricular function, several imaging techniques have been developed to assess myocardial deformation in the context of its very complex architecture [1–3]. The left ventricular (LV) myocardial architecture is organized into three distinctive layers: (1) the subendocardial layer, formed by fibres oriented longitudinally from base to apex; (2) the mid-wall layer, with circumferentially oriented fibres; and (3) the subepicardial layer where fibres are longitudinally oriented but directed from the apex to the base. Due to this complex architecture, in systole, the LV deforms along different directions determining longitudinal and circumferential shortening, radial thickening and torsion (Fig. 1).

## Definition of myocardial strain and strain rate

Myocardial strain (MS) measures the degree of deformation of a myocardial segment from its initial length (L0, usually in end diastole) to its maximum length (*L*, usually in end systole) and is expressed as a percentage. It is determined by the following formula:

$$ \mathrm{MS}:\mathrm{L}\hbox{-} \mathrm{L}0/\mathrm{L}0 $$

There are various definitions of strain: (1) Lagrangian^1^ strain, in which displacements are calculated at a fixed material point in the myocardium using the deforming myocardium itself as a reference, and (2) Eulerian^2^strain, which represents the tissue strain at a specific location in space, so spatial coordinates are fixed, but material points keep changing. Imaging modalities are mainly based on the analysis of Lagrangian strain [4, 5].

Following the different directions in which the myocardium deforms, longitudinal, circumferential and radial strain can be calculated. *Longitudinal strain* represents the longitudinal shortening from the base to the apex, and it is expressed by negative values. *Radial strain* is the radially directed myocardial deformation towards the centre of the LV cavity and indicates the LV thickening and thinning motion during the cardiac cycle; it is expressed by positive values. *Circumferential strain* derives from LV myocardial fibre shortening along the circular perimeter observed on a short-axis view, and it is consequently represented by negative values. Strain rate represents the rate at which those deformations occur [6, 7]. LV torsion is created by the clockwise rotation of the basal segments and the counter-clockwise apical rotation relative to a stationary mid-myocardial reference point [8] and is directly related to the circumferential-longitudinal shear angle [9] (Table 1).

**Table 1:** Definition of principal myocardial deformation measures

	Definition	Value
Longitudinal strain (%)	Longitudinal base-apex shortening	Negative
Circumferential strain (%)	Shortening along the circular perimeter	Negative
Radial strain (%)	Thickening myocardial deformation towards the centre of the LV cavity	Positive
Strain rate	Rate of shortening of a length	1/s
Torsion	Wringing motion of the ventricle around its long axis	Degrees

## Cardiovascular magnetic resonance techniques to assess myocardial deformation

Cardiovascular magnetic resonance (CMR) has emerged as the reference standard for the evaluation of biventricular morphology and function. In the last decades, several CMR techniques have been developed to measure cardiac muscle motion and deformation [10–13]. From a general point of view, they can be divided into techniques based on tailored image acquisition and post-processing methods (Table 2).

**Table 2:** Characteristics of CMR techniques to assess myocardial strain with main advantages and disadvantages

CMR technique	How do they work	Advantages	Disadvantages
Acquisition methods			
CMR tagging	Tracks magnetization tags	• Good tags tracking • Better reproducibility • Many validation studies	• Low spatial resolution • Tag fading • Long post-processing time • Low temporal resolution Long acquisition time
PVM	Encodes myocardial velocity, in the tree directions, in the phase of the signal		
DENSE	Encodes tissue displacement into the phase of an image	• Good-quality strain in short acquisition time	• Low signal-to-noise ratio • Modest clinical experience
SENC	Uses magnetization tags parallel to the image plane combined with out-of-plane phase-encoding gradients	• Quick post-processing needed	• Tag fading • Modest clinical experience • Radial strain non-measurable
Post-processing method			
CMR-FT (TT)	Tracks features in the image and recognizes them in the successive image of the sequence	• No additional image acquisition • Post-processing approach on existing data	• Through-plane motion artefacts • Limited by pixel size • No standardization

*PVM* phase velocity mapping, *DENSE* displacement encoding with stimulated echoes, *SENC* strain-encoded imaging, *FT* feature tracking, *TT* tissue tracking

### Strain acquisition methods

#### Cardiovascular magnetic resonance tagging

CMR tagging was first introduced by Zerhouni et al. [10]. It consists of a preparation phase in which magnetic labels (black lines, tags) are orthogonally superimposed to the myocardium at the beginning of a cine sequence. The subsequent deformation of those lines throughout the cardiac cycle is analysed [14, 15]. A specific radiofrequency pre-pulse, spatial modulation of magnetization (SPAMM), was developed to efficiently saturate parallel planes throughout the imaging volume, and later, complementary SPAMM (CSPAMM) improved the contrast, providing sharper and better defined lines [16]. CSPAMM can be applied twice in two orthogonal directions creating a grid pattern. Since magnetization is a characteristic of the tissue, the tags move together with the myocardium; therefore, by tracking the motion of the tags, myocardial deformation parameters can be assessed [14]. The tags will gradually fade during the cardiac cycle because of tissue T1 relaxation and the imaging radiofrequency pulses. That limits the use of tagging to the first two thirds of the cardiac cycle, mining the assessment of some regional myocardial abnormalities and of diastolic function.

Visual assessment of tag movement provides immediate information on regional wall motion abnormalities (WMA). However, for quantitative analysis, several post-processing semi-automated methods have been developed. The FINDTAGS software is based on the detection and tracking of tag lines in the image. Optical flow techniques are based on the tracking of pixels from frame to frame based on constant brightness, which means that they identify the brightness of a pixel and then look for a nearby pixel that has a similar signal intensity. Harmonic phase (HARP) analysis tracks pixels from frame to frame, based on a constant phase; due to its almost fully automated nature, HARP is the most widely used method [17]. Although CMR tagging is the most validated CMR technique to assess myocardial strain [18–20], it is affected by tag fading and low spatial resolution (limited by the number and density of tag lines), which reduce its accuracy when applied to thin walls (as it does not allow for optimal tag spacing) [15], and requires long post-processing time, although this is reduced by HARP analysis.

#### Phase velocity mapping

Phase velocity mapping (PVM) was first used to measure velocities inside the heart, more than 30 years ago [21]. It is based on the use of a bipolar gradient, which encodes the velocity in the phase of the signal. This technique is widely available as a standard sequence, it has quick post-processing, and it is commonly used to estimate valvular flows. Myocardial deformation parameters are extracted from the myocardial velocity in the three directions, by calculating the spatial derivatives of the velocities at each pixel. PVM has high spatial resolution, as it is not affected by tag number. Separate breath-holds were initially required for each direction of encoding; then, the development of segmented sequences enabled PVM data to be acquired, in the three directions, within a single breath-hold [22, 23]. Although respiratory navigator gating has improved temporal and spatial resolution [12], temporal resolution is still lower than that with tagging [5] and at the cost of increased acquisition time.

#### Displacement encoding with stimulated echoes

Displacement encoding with stimulated echoes (DENSE) is a technique that encodes tissue displacement into the phase of an image. It consists of three radiofrequency pulses used to generate a stimulated echo, while gradients encode displacement into the signal phase [5]. The sequence can be repeated with encoding in orthogonal direction to obtain a two-dimensional displacement for each pixel. Fast cine DENSE pulse sequence was developed based on the echo combination reconstruction with intrinsic phase correction, using balanced steady-state free precession (b-SSFP). This sequence has been proved to provide good-quality strain within a reasonable breath-hold duration [11, 24]. However, being based on stimulated echo, it is characterized by low signal-to-noise ratio (SNR), and like with tags, the encoding tends to disappear through the cardiac cycle due to T1 relaxation time [25].

#### Strain-encoded imaging

Strain-encoded (SENC) imaging is a special modification of the CMR scanner software that enables the quantification of regional deformation of tissue. To calculate myocardial strain, SENC uses magnetization tags parallel to the image plane (not orthogonal as in CMR tagging) combined with out-of-plane phase-encoding gradients along the selected direction [26, 27]. Therefore, two- and four-chamber views are generated to calculate circumferential strain, and longitudinal strain is measured from short-axis images, while radial strain cannot be measured. Through-plane strain is directly related to the pixel intensity in the resulting images, and limited post-processing is needed. However, the tags still fade due to T1 relaxation time and therefore SENC cannot be used to assess myocardial deformation throughout the entire cardiac cycle [13].

### Post-processing cardiovascular magnetic resonance technique to assess myocardial strain

#### Feature tracking

**Fig. 2:**
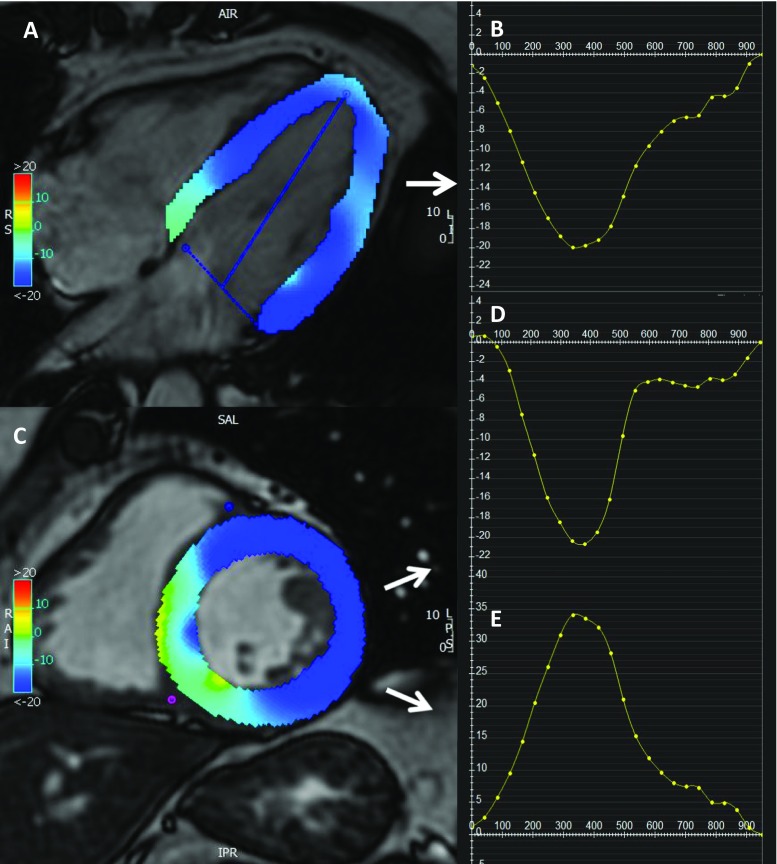
Example of coloured strain analysis with a feature-tracking software (Circle CVI42®). From long-axis four-chamber SSFP cine image (**a**), longitudinal strain curve is derived (**b**) and short-axis SSFP image (**c**) is used for calculation of circumferential (**d**) and radial strain curves (**e**)

Feature-tracking technology is a post-processing method that can be applied to routinely acquired cine CMR images. It is an optical flow [28] method, and it is based on identifying features in the image and tracking them in the successive images of the sequence [29, 30]. This way, the displacement of myocardial segments can be measured. More precisely, it is based on defining small square windows, centred around a feature, on a first image and searching the “as-much-as-possible similar” greyscale pattern on the following image [31]. The features tracked by CMR-feature tracking (FT) are anatomic elements that are different along the cavity-myocardial tissue boundary, and they are found by methods of maximum likelihood in two regions of interest between two frames [32]. The CMR-FT software’s automatic border tracking starts after manually defining endocardial and epicardial borders (excluding papillary muscles and trabeculae) and the mitral valve annular plane at end diastole. It estimates global longitudinal strain from two long-axis SSFP cine images while circumferential and radial strains are derived from the short-axis cine images (Fig. 2). Artefacts due to through-plane motion are the main limitations, as features moving out of plane cannot be tracked [33, 34]. FT was developed for two-dimensional images; however, this technology can be applied to track three-dimensional regions. Indeed, performing a three-dimensional tracking allows for the detection of all the deformation parameters simultaneously, reducing artefacts from through-plane motion. However, experience with three-dimensional applications is still limited [29]. Furthermore, CMR-FT is based on the assumption that the deformation measured derives from the myocardium and that the blood motion does not interfere with it. However, blood motion can affect the tracking close to the endocardial regions, where unrealistic results may be noticed. Finally, CMR-FT is limited by the pixel size; displacement of less than the pixel size may not be detected [4].

#### Feature tracking normal values and reproducibility

Many studies have provided normal values for myocardial strain assessed with CMR-FT (Table 3), showing significant gender-related differences in the longitudinal strain [35], which is greater in females, and a degree of correlation between age and radial and circumferential strain values [36, 37]. In general, circumferential and longitudinal strain values higher than −17 and −20%, respectively, are considered pathological [38]. All tracking techniques have proved to be more robust and reproducible for global strain values as compared to regional ones, with the most consistent parameters being global longitudinal and global circumferential strain [35, 37, 39]. Among strain values assessed with FT, global circumferential strain has the best interobserver agreement, while global radial strain has the worst one showing large ranges between studies [38, 40]. The poor tracking of the basal segments due to the complex architecture of the mitral annuls is probably responsible for the worse consistency of global longitudinal strain values. Variability of strain values between different studies mainly comes from the existence of different vendors with lack of contouring standardization. Schuster et al. [40], in a recent study comparing strain values measured in the same patients with different vendors (TomTec and Circle cardiovascular imaging Tissue-Tracking, TT, software), showed that TT provided lower values of circumferential strain and torsion. They also highlighted a better reproducibility for torsion and circumferential strain values when assessed with TomTec, while Circle showed less variability for radial strain.

**Table 3:** Studies providing normal CMR feature-tracking LV strain values in the three different directions

Study	*N*	GLS (%)	GCS (%)	GRS (%)
Augustine et al. [35]	145	−19 ± 3	−21 ± 3	25 ± 6
Andre et al. [36]	150	−23.4 ± 3 (endocardial) −21.6 ± 3 (myocardial)	−27.2 ± 4 (endocardial) −21.3 ± 3 (myocardial)	36.3 ± 8
Morton et al. [39] (average of 3 scans)	16	−20.5 ± 5	−17.4 ± 4	20.8 ± 6
Taylor et al. [34]	100	−21.3 ± 5	−26.1 ± 4	39.8 ± 8

*GLS* global longitudinal strain, *GRS* global radial strain, *GCS* global circumferential strain

#### Acquisition vs post-processing methods

Advantages of CMR-FT are mainly related to its post-processing nature as (1) it does not require additional image acquisition time in the scanner, (2) it can be easily applied to all CMR routine scans as it uses standard SSFP cine CMR images, normally acquired to measure biventricular volumes, and (3) it is rapid and semi-automated, with a short post-processing time. However, as previously stated, it is subjected to through-plane motion artefacts, and a good tracking requires high image quality with adequate spatial and temporal resolution [29]. CMR tagging has been the most validated CMR technique to assess myocardial deformation, and although the methods to track the tags are similar to those used for tissue tracking, the imposed tags are easier to follow than the natural features, allowing for better reproducibility [35, 41–43]. Suboptimal spatial resolution, together with tag fading during the cardiac cycle, is tagging’s main pitfalls. DENSE provides good endocardial border definition and high spatial resolution images, but poor clinical and research experience limited its use, as happened to SENC. From a general point of view, dedicated acquisition time is what mainly affects the use of an acquisition method in routine clinical practice; hence, tissue tracking is now the preferred technique to assess myocardial deformation parameters.

## Comparison with other imaging modalities

Speckle tracking echocardiography (STE) has been considered an accurate and convenient method to assess myocardial strain, being easy to perform and largely available [44]. It is based on the same principle described for tissue tracking. However, in STE, the software tracks tiny echo dense speckles within the myocardium, and although it is mainly a post-processing method, it still requires a specific frame rate (50 to 70 frames/s) during image acquisition and high image quality [45]. STE provides non-Doppler, angle-independent, and objective quantification of myocardial deformation. However, its widespread applicability may be hindered by poor acoustic windows and, considering that speckle-tracking echocardiography is based on single-cardiac-cycle strain analysis, it is not possible to conduct a myocardial deformation study in patients with arrhythmias [46]. As for CMR-FT, STE can be affected by through-plane motion artefacts, but this issue can be addressed using 3D STE. Many studies have compared myocardial strain parameters measured with STE and CMR FT, and they underlined a good agreement between the two techniques [47–50]. Global longitudinal strain appeared to be more accurate with STE, while, as previously stated, global circumferential strain showed a better reproducibility if calculated with CMR FT. Recently, TT software has been applied to computed tomography (CT) images, showing not only the feasibility of the strain analysis but also a higher number of successfully analysed segments compared to two-dimensional echocardiography [51]. However, more studies are needed to validate strain values measured from CT images.

## Clinical application

Clinical application of myocardial strain imaging has been increasingly assessed over the past few years.

### Ischemic heart disease

The application of myocardial deformation has been widely studied in ischemic heart disease (IHD). All myocardial strain components are impaired in infarcted territories, as compared to both adjacent and remote territories [25, 52, 53], and myocardial strain is inversely related to area at risk [53], infarct size and infarct transmurality [52–54]. Segmental analysis of myocardial strain allows to distinguish areas of subendocardial from areas of transmural infarction [25], which typically show more impaired strain values. Strain impairment is more evident than wall thickness alteration in infarcted tissue, and analysis with myocardial tagging has proved to be more accurate than wall thickness analysis in the identification of infarcted areas [15]. The most recent FT technique has been validated against the gold standard myocardial tagging, and its robustness was tested for both global and segmental strain analyses after acute myocardial infarction (MI) [53].

Segmental analysis of strain also allows the identification of myocardial segments that will recover in function after an acute ischemic event [25, 52, 53]; in a follow-up study on 74 patients after acute MI, reduced circumferential strain was associated with recurrent MI and need for repeated revascularization [55]. Conversely, a recent study comparing tagging and CMR-FT following acute MI failed to show a predictive value of strain in determining reverse LV remodelling at follow-up, despite showing good correlation between all strain components and infarct size at baseline [54].

An emerging role of strain is in the assessment of myocardial ischemia. Dobutamine stress CMR (DS-CMR) with the addition of tagging has shown to be superior to DS-CMR without tagging in the detection of new regional WMA [56]. Analysis of WMA is based on visual assessment, thus being operator-dependent; strain analysis based on CMR-FT has shown to be a reliable tool to reduce observer dependency [57]. As WMA develop late in the ischemic cascade, myocardial strain analysis allows earlier detection of ischemia-induced myocardial dysfunction: a recent FT study [58] showed that, despite no differences at rest, at high-dose dobutamine, circumferential strain was significantly impaired in territories supplied by severe coronary artery stenosis, as compared to both normal territories and those supplied by non-significant coronary artery stenosis, and these findings were confirmed also at intermediate-dose dobutamine: the earlier detection of ischemia provided by strain analysis might prevent the need of high-dose stress testing, thus reducing all related risks.

### Non-ischemic heart disease

Myocardial strain is impaired also in non-ischemic heart disease (NIHD). Circumferential shortening is less than a third in patients with dilated cardiomyopathy (DCM) as compared to healthy volunteers [15]; as mid-wall fibres mostly contribute to circumferential strain, the presence of mid-wall fibrosis in NIHD has shown to impair circumferential strain, while longitudinal and radial strains seem not to be affected [59]. The analysis of myocardial strain offers new insight into disease’s mechanisms: intramural functional abnormalities have been shown to extend beyond the presence of late gadolinium enhancement (LGE) in patients with hypertrophic cardiomyopathy (HCM), as intramural systolic strain is abnormal in hypertrophied segments as compared to segments without hypertrophy, irrespective of the presence of LGE [60]. However, a linear correlation between myocardial strain and the amount of LGE has been shown in different studies [15, 61, 62], both at the global and segmental levels, so that it has been inferred that strain analysis might be considered in the future to indirectly detect the presence of scar, without need of contrast agent [62].

Myocardial strain was impaired in patients with clinically diagnosed acute myocarditis and no other abnormal findings on CMR and in patients with CMR findings of myocarditis but preserved left ventricular ejection fraction (LVEF) [63, 64]. Chemotherapy-induced cardiotoxicity by means of abnormal strain is impaired well before the decline in LVEF [65].

Finally, myocardial strain might play a future role in tailoring treatment. Patients with severe aortic stenosis have impaired myocardial strain, irrespective of symptom severity, which is one of the criteria to time surgery [66]; therefore, myocardial deformation analysis might be a novel marker to select the best timing to surgery. It is well known that cardiac resynchronisation therapy (CRT) is associated with a non-negligible proportion of non-responders and increasing efforts have been made over the years to select the best candidates to CRT; in a recent study by Taylor et al. [67], CMR has been used to detect the presence of myocardial scar and the latest mechanical myocardial activation as assessed by strain: lead positioning on the site of latest mechanical activation, in the absence of myocardial scar, not only improved LV reverse remodelling but was also associated with lower mortality, thus showing that the implementation of myocardial deformation analysis could be a promising tool to increase patients’ response to CRT.

## Right ventricular strain imaging and clinical applications

The right ventricle has a complex anatomy (highly trabeculated, with a thin wall) and function which makes it difficult to assess [68–70]. CMR has become the gold standard for the assessment of right ventricular (RV) volumes and function [68], and it was also the first method to provide multi-directional RV strain analysis: early studies of CMR tagging with SPAMM tags showed that the analysis of RV segmental function was feasible and reproducible [70] and it unravelled the non-uniform mechanics of the right ventricle, characterized by a basal to apical gradient of RV free wall deformation, with increased strain values at the apex. These results were confirmed using 3D cine DENSE imaging in a small study on five healthy volunteers [71]. Limits related to tagging are due to the assessment of the thin-walled right ventricle. SENC gets all strain values from a single four-chamber view, allowing higher spatial resolution and subsequent better RV endocardial delineation [72], with the assessment of RV free wall circumferential strain with low inter- and intraobserver variability.

**Fig. 3:**
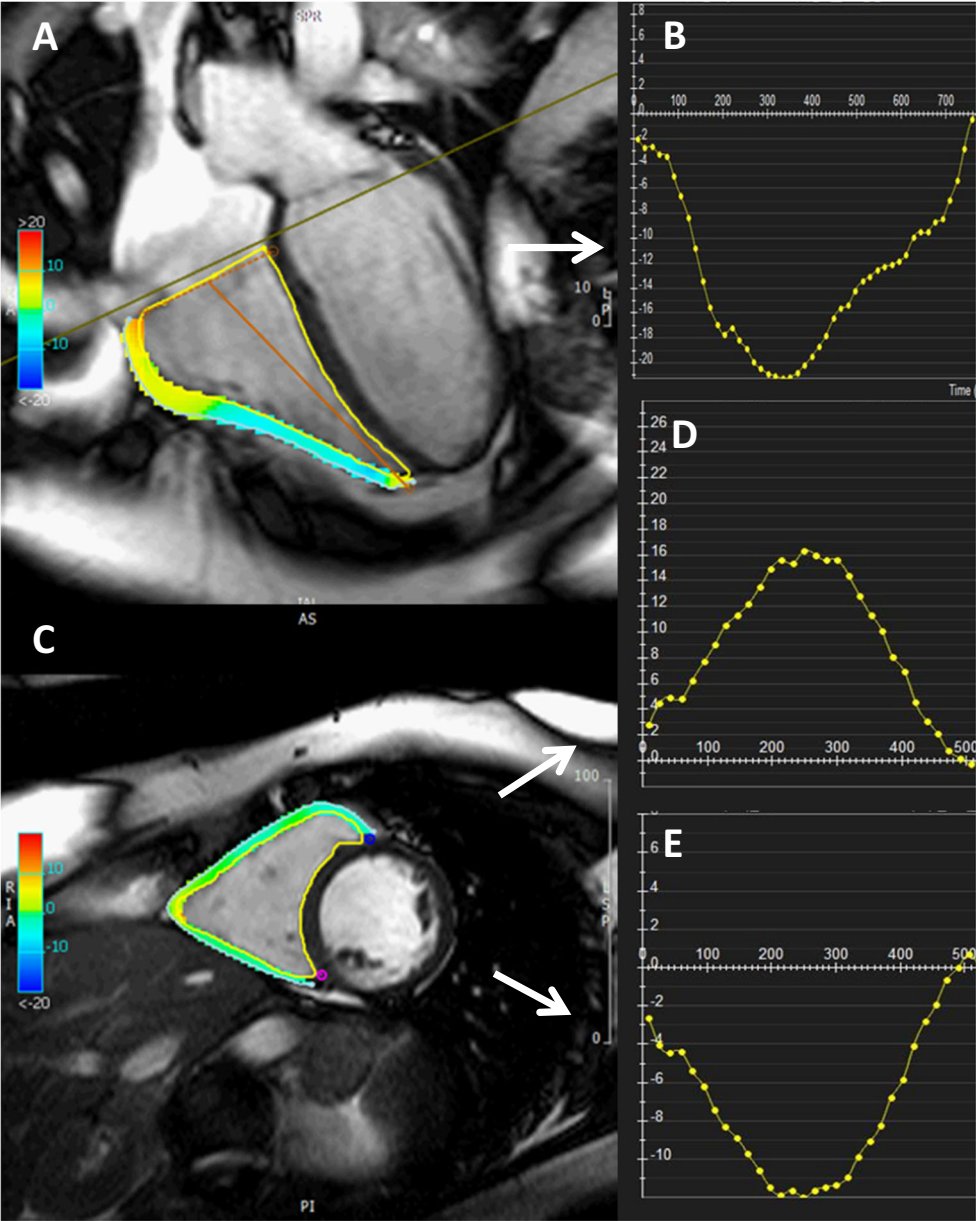
Example of coloured strain analysis with a feature-tracking software (Circle CVI42®) for the calculation of RV myocardial strain. **a** Four-chamber SSFP cine image, used to derive the RV longitudinal strain curve (**b**). **c** Short-axis SSFP cine image allows for the calculation of radial (**d**) and circumferential (**e**) RV strain curves

Similar to what it has been observed for LV strain, normal values of RV strain differ slightly between different studies, as a consequence of the different techniques used for the analysis. A recent study used the newer FT technique to determine normal values for RV myocardial strain; it estimates radial and circumferential strain from the short-axis images and longitudinal strain from cine long-axis images (Fig. 3; [73]): similarly to the left ventricle, female showed to have higher peak circumferential basal and mid-cavity strain values, with no correlation found between myocardial strain and age and body mass index, and the apex showed the highest circumferential strain values, as compared to base and mid-cavity. Using FT, mid-cavity peak circumferential strain offered the best interobserver reproducibility, while the worst reproducibility was found for basal and mid-cavity circumferential strain.

The availability of software that allowed the assessment of RV strain prompted its application in diseases that typically target the RV, such as congenital heart disease (CHD), pulmonary hypertension (PH) and arrhythmogenic right ventricular cardiomyopathy (ARVC).

CMR-FT has been used in patients with repaired tetralogy of Fallot (rTOF) and compared to STE [74, 75], showing to provide comparable results for both ventricles and to have superior interobserver agreement with regard to longitudinal RV global strain. In the same study, strain values showed to be reduced in rTOF patients and to be related not only to systolic contraction (LVEF and RV ejection fraction (RVEF)), but also to patient’s functional capacity at the cardiopulmonary exercise test.

CMR tagging with SPAMM showed that all myocardial strain components are reduced in PH patients [70]. The RV fibres are predominantly arranged along the longitudinal axis, so that longitudinal shortening mostly contributes to RV shortening in healthy volunteers [70, 76]; however, contribution of circumferential strain is believed to become more important in PH patients. RV strain was impaired in PH patients and related to disease severity [76]. Similar to findings in the left ventricle, a linear correlation between the amount of LGE and the degree of strain impairment has been found in PH patients.

The assessment of both global and segmental RV functions is pivotal for the multi-parametric diagnosis of ARVC, and RV myocardial strain has proved to be an extremely useful tool [77–79]. RV global and segmental strains were significantly impaired in ARVC patients, independent of RV dimensions and function, so that impairment of RV strain might represent an earlier marker of the disease [78]; another study showed that RV strain is impaired in overt ARVC, although it did not show a significant difference in pre-clinical ARVC patients [79].

## Atrial strain

Atrial function has been more and more recognized as an important element in many cardiac diseases, especially those characterized by high filling pressure and diastolic dysfunction. It consists of three components: reservoir function (collection of blood from pulmonary veins), conduit function (during the passage of blood to the left ventricle) and contractile pump function (atrial contraction in the late diastole). Global measures, such as volumes and areas, are not enough to describe this complex function [80, 81], and tissue magnetization techniques cannot be applied to assess atrial deformation due to the thin atrial walls. Recently, TT technology has been used to assess left atrial (LA) function. As for ventricular strain assessment, LA endocardial borders are manually traced in the two- and four-chamber views, when the atrium is at its minimum volume after atrial contraction and the automated tracking algorithm is applied. Normal values for LA-global strain are 29 ± 5% for the reservoir, 21 ± 6% for the conduit, and 8 ± 3% for the atrial contraction phases. Strain values during atrial contraction tend to increase in elderly subjects consistent with physiology of normal ageing [80, 81]. Feasibility and reproducibility of CMR-FT-derived strain parameters from both atria have also been assessed, showing promising preliminary results [82].

## Prognosis

The analysis of myocardial deformation has shown to provide important prognostic information. A study on more than 500 patients referred to CMR for suspected cardiomyopathy, most of whom had no cardiovascular risk factors, showed that LVEF, the presence of LGE and impaired global circumferential strain were all associated with a worse outcome (all-cause mortality, hospitalization for heart failure and sudden cardiac death (SCD)) [83]; more interestingly, presence of LGE and impaired circumferential strain were independent predictors of adverse outcome, irrespective of LVEF. A similar study performed in 210 patients with reduced LVEF (<50%) but without history of coronary artery disease, infarction or hypertension showed that impaired strain predicted the end point of cardiac death, heart transplant and ICD discharge, with impaired longitudinal strain being the strongest predictor of adverse outcome, irrespective of both the presence of LGE and LVEF [84]; on the other hand, patients with preserved longitudinal strain showed a good outcome, irrespective of LV dysfunction or presence of LGE. Similar results were observed in patients with HCM, where impaired radial and longitudinal strain were associated with worse outcome [85].

Assessment of both LV and RV myocardial strain has shown to be a useful tool to predict major adverse cardiovascular events (MACE); in a FT study on more than 300 patients, adding biventricular strain analysis to conventional ejection fraction assessment increased the detection of patients experiencing adverse outcome: LV global transverse strain and RV global radial strain proved to be the strongest predictors of MACE [86].

Finally, in large populations of patients with repaired TOF, longitudinal and circumferential strains were associated with NYHA class, while bi-ventricular systolic function was not able to stratify patients according to their symptoms; moreover, both LV circumferential strain and RV longitudinal strain were predictors of adverse arrhythmic outcome [87, 88].

## Future directions

Many CMR modalities have been developed to assess myocardial deformation, and the introduction of newer CMR software that allow myocardial deformation analysis based on post-processing methods, with no need to acquire extra pictures, has prompted the clinical application of myocardial strain imaging. However, in order to allow exclusion from the analysis of segments with suboptimal image quality, standardization in the manual contouring and automated evaluation of tracking quality is needed. Similarly, agreement between different vendors is mandatory, to ensure a comparable technological ground between different studies. Therefore, the limitations of each technique need to be considered before the data can be used in the clinical decision-making process.

## Footnotes

1 It is named after Giuseppe Ludovico Lagrangia, an Italian mathematician, who formulated different theories; among them, there is the Lagrangian mechanic which is used in the strain analysis.

2 The Eulerian mechanic is more used in fluid dynamic.
